# The Effects of Tetracycline Residues on the Microbial Community Structure of Tobacco Soil in Pot Experiment

**DOI:** 10.1038/s41598-020-65203-w

**Published:** 2020-05-29

**Authors:** Jiayu Zheng, Jixu Zhang, Lin Gao, Fanyu Kong, Guoming Shen, Rui Wang, Jiaming Gao, Jiguang Zhang

**Affiliations:** 1 Tobacco Research Institute of Chinese Academy of Agricultural Sciences, Qingdao, 266101 P.R. China; 2 Tobacco Company of Hubei Province, Wuhan, 430030 P.R. China; 3 Kunming Tobacco Company, Kunming, 651500 P.R. China

**Keywords:** Microbial ecology, Microbial ecology, Environmental impact, Environmental impact

## Abstract

To evaluate the micro-ecological effects of tetracycline residues on tobacco soil, high-throughput sequencing technology was used to study the effects of the addition of different concentrations (0, 5, 50, and 500 mg·kg^−1^) of tetracycline on the abundance, diversity, and structure of bacterial and fungal communities in the rhizosphere and non-rhizosphere soil of flue-cured tobacco in China. Results showed that the presence of tetracycline had an important but varying effect on soil bacterial and fungal community richness, diversity, and structure. Changes in the diversity indices (Chao index and Shannon index) of soil bacterial and fungal communities showed a similar pattern after the addition of tetracycline; however, a few differences were found in the effects of tetracycline in the rhizosphere and non-rhizosphere soil, suggesting an evident rhizosphere-specific effect. The bacterial community at the phylum level in the rhizosphere closely clustered into one group, which might be the result of tobacco root secretions and rhizodeposition. Tetracycline showed a concentration-dependent effect on the soil bacterial community structure. The soil bacterial community structures observed after treatments with higher concentrations of tetracycline (50 and 500 mg·kg^−1^) were found to be closely related. Moreover, the effects of the treatments with higher concentrations of tetracycline, on the soil bacterial community at the phylum level, were different from those with lower concentrations of tetracycline (5 mg·kg^−1^), and CK treatments. This might have resulted from the induction of increasing selective pressure with increasing antibiotic concentration. Tetracycline continued to affect the soil bacterial community throughout the experiment. Tetracycline was found to have a varying impact on the community structure of soil fungi compared to that of soil bacteria, and the addition of an intermediate concentration of tetracycline (50 mg·kg^−1^) significantly increased the soil fungal diversity in the non-rhizosphere soil. The biological effects of tetracycline on the soil fungal community and the fungal-bacterial interactions, therefore, require further elucidation, warranting further research.

## Introduction

Tetracycline antibiotics are broad-spectrum antibiotics and include tetracycline, oxytetracycline, chlortetracycline, and semi-synthetic derivatives such as methicillin, doxycycline, dimethylaminotetracycline, etc. Tetracycline antibiotics are widely used due to their low cost and broad-spectrum bactericidal properties. China is a major producer and consumer of tetracycline antibiotics. In 2008, the export volume of tetracycline antibiotics reached 1.34 × 10^7^ kg. Further, tetracycline is the most used antibiotic in the livestock and poultry industries in China^1^. Tetracycline antibiotics that enter the animal are not entirely absorbed, and about 30–90% of them is excreted as a parent compound^2^. The residual antibiotics in animal waste are often transferred into agricultural soils as fertilizer^3^. Tetracycline antibiotics enter the soil environment through a variety of pathways and have negative impact on the soil ecosystem as well as human health through a series of physical, chemical, and biological processes^4,5^. Tetracyclines are commonly detected in agricultural soils at concentrations of 5–25 mg^.^kg^−1^ in China^6^. In addition, long-term use of tetracycline antibiotics can induce microbial resistance and have potential environmental impacts^7,8^. Therefore, the potential ecological hazards associated with antibiotic pollution of soils have become a hot topic in the field of agriculture.

The microbial community present in soil forms the basis for its ecological function, which is closely related to the circulation of soil nutrients, the improvement of soil fertility, the degradation of soil pollutants, and the growth and disease control of crops^9,10^. In contrast to plant diversity, the meso- and macrofauna, soil microbial composition and diversity are rather newly recognised aspects. A major problem in soil microbial analysis has been that classical microbial culture techniques cannot be used for the characterisation of most soil microorganisms. Further, some methods including the Biolog system, biomarker methods, restriction fragment length polymorphism (RFLP), fluorescence *in situ* hybridisation (FISH), stable isotope probe (SIP), and other molecular biology techniques have certain limitations in the study of soil microbial community structure, and are unable to clarify the microbial community structure in detail^11^. With the advancement of modern molecular biotechnology, a new generation of high-throughput sequencing technology has emerged as a powerful technique over the last years. It can be used for library construction and sequencing, followed by the study of microbial community structure at a higher level^12^. MiSeq is the second generation of high-throughput sequencing platforms, developed by Illumina in 2011. It uses an indexing strategy and overlapping 2 × 150 bp reads, generating more than 1000 Mb of data per run. With the development of the MiSeq platform’s dual-end PE250 and PE300 sequencing strategies, the read length has increased, which has considerably improved the accuracy of species diversity identification^13^. Sequencing one or more hypervariable regions of 16 S rDNA using the MiSeq platform has the characteristics of high sequencing depth, identification of low-abundance community species, and low cost. Thus, it has become the first choice for studying microbial community diversity^14^. Further, it has been widely applied in the study of microbial community diversity in environmental samples^12,14,15^.

Tobacco, as one of the most important industrial crops, is widely grown in China^16^. During the cultivation of tobacco, the application of organic fertilizers, such as livestock manure, farm manure, and commercial organic fertilizers, could easily lead to tetracycline pollution of tobacco soil. However, how about the ecological effects of tetracycline pollution on microorganisms in tobacco soil? There have been a few studies focused on the comparison of the effects of tetracycline on soil bacterial and fungal community structure in the rhizosphere and non-rhizosphere soil of flue-cured tobacco. In the current study, we used high-throughput sequencing technology to explore the effects of different concentrations of tetracycline on the bacterial and fungal community structure of the tobacco rhizosphere and non-rhizosphere soil in a pot experiment.

## Materials and methods

### Site description and soil sampling

Pot experiments were conducted in 2014 at the Wangchengpo Modern Tobacco Agricultural Science and Technology Park in Enshi City, Hubei province, China.

The soil for the pot experiments was collected from the forest land near the long-term tobacco planting field of the village of Mao bacao, Baiguo town. Tetracycline antibiotics were not detected in the potting soil, which was analysed by liquid chromatography in combination with tandem mass spectrometry^17^. The main characteristics of the soil before the start of the experiment were as follows: pH 6.9, soil organic carbon 19.23 g·kg^−1^, alkali-hydrolysable N 85.37 g·kg^−1^, available phosphorus (Olsen-P) 62.70 g·kg^−1^, extractable K 218.67 g·kg^−1^. Soil type was yellow-brown soil. The soil was air-dried, sieved to a diameter of 2 mm, mixed with chemical fertilizers (4 g N per pot and N: P_2_O_5_: K_2_O = 1:1.5:3, in line with the amount of fertilizer applied in the local tobacco planting area). Each plastic pot was filled with 15 kg of sieved soil and enough water was added to reach 60% water holding capacity (WHC). The four treatments in the pot experiment were as follows: CK treatment (no tetracycline addition); T1 treatment (5 mg Tc kg^−1^ dry weight soil); T2 treatment (50 mg Tc kg^−1^dry weight soil); T3 treatment (500 mg Tc kg^−1^dry weight soil). There were three replicates per treatment. Yunyan 87 was chosen as the tested flue-cured tobacco. The test tetracycline hydrochloride (purity 99.5%) was obtained from National Institutes for Food and Drug Control.

Tetracycline was dissolved in distilled water and then poured evenly into each pot after the tobacco seeding had been transplanted into the pots on May 20th. The same amount of distilled water was added to the pot in CK treatment. During the entire experimental period, water was added every 3–5 days to maintain the soil moisture of each pot at about 60% WHC.

The soil strongly adhering to the roots and within the space explored by the roots was considered the rhizosphere soil^18^. Rhizosphere soil (R-soil) samples were collected by the root-shaking method at the mature stage of tobacco (5th September) in 2014. Meanwhile, the non-rhizosphere soil (N-soil) samples were collected from five points in each pot, mixed, and homogenised to obtain about 0.5 kg. Both R-soil (RCK, RT1, RT2, RT3) and N-soil (NCK, NT1, NT2, NT3) samples were collected and stored at −80 °C for further analyses of the soil microbial community.

### DNA extraction

Total genomic DNA was extracted from soil samples via the Power Soil DNA extraction kit (MOBIO, USA), following the manufacturer’s instructions. DNA concentration and purity were monitored on 1% agarose gels.

### PCR amplication and high-throughput sequencing

Prepared DNA samples were sent to Novogene Bioinformatics Technology Co., Ltd (Beijing, China) for further analysis. DNA was diluted to 1 ng/μl using sterile water. Primers used were: 16 S V4: 515F-806R, 18 S V4: 528F-706R, ITS1: ITS1F- ITS2. 16 S/18 S rRNA genes were amplified using these specific primers. The PCR mixture (30 μL) contained 10 ng template DNA, 15 μL of Phusion® High-Fidelity PCR Master Mix (New England Biolabs), 0.2 μM of forward and reverse primers. The PCR program consisted of 1 min at 98 °C, followed by 30 cycles at 98 °C for 10 s, 50 °C for 30 s, 72 °C for 60 s, and, finally, 72 °C for 5 min. The same volume of 1X loading buffer (containing SYBR Green I) was mixed with PCR products, and electrophoresis on 2% agarose gel was performed for detection. Samples with a bright main strip between 400–450 bp were chosen for further analysis. PCR products were mixed in equidensity ratios and purified with the Gene JET Gel Extraction Kit (Thermo Scientific). Sequencing libraries were generated using NEB Next® Ultra™ DNA Library Prep Kit for Illumina (NEB, USA) following the manufacturer’s recommendations, and index codes were added. The library quality was assessed on the Qubit@ 2.0 Fluorometer (Thermo Scientific) and the Agilent Bioanalyzer 2100 system. The library was sequenced on an Illumina MiSeq platform, and 250 bp/300 bp paired-end reads were generated.

### Pyrosequencing data processing

Sequences were processed using the Quantitative Insights in Microbial Ecology (QIIME) pipeline v.1.3.0^19^. Paired-end reads from the original DNA fragments were merged using FLASH^20^. Paired-end reads were assigned to each sample according to their unique barcodes. Sequence analysis was performed by the UPARSE software package using the UPARSE-OTU and UPARSE-OUTref algorithms. In-house Perl scripts were used to analyse alpha (within samples) and beta (among samples) diversity. Sequences with ≧97% similarity were assigned to the same OTUs^21^. We picked a representative sequence for each OTU and used the RDP classifier to annotate taxonomic information for each representative sequence. In order to compute alpha diversity, we rarified the OTU table and calculated two metrics: the Chao index estimates the species abundance; the Shannon index estimates the diversity of the community.

Graphical representation of the relative abundance of bacterial and fungal diversity from the phylum to the species level could be visualised using the Krona chart. We used the unweighted pair group method with Arithmetic mean (UPGMA) clustering^21^. UPGMA Clustering is a type of hierarchical clustering method using average linkage and can be used to interpret the distance matrix.

### Alpha and beta diversity

Observed species richness, Chao index, phylogenetic distance, and the Shannon index were computed in QIIME. Beta diversity was determined using the UniFrac distance metric^22^.

## Results

### Analysis of high-throughput sequencing results and alpha diversity of the community

High-throughput sequencing of the bacterial community revealed that an average of 12,922 sequences and 515 OTUs per sample were obtained from all soil samples (Table 1). The largest number of soil bacteria sequences was obtained from T3, and the smallest was from T1 among all treatments of R-soil, following filtering and chimera removal. The highest number of soil bacteria sequences was found in T2, while the lowest was in T3 amongst all treatments of N-soil. Furthermore, OTU quantities among all treatments in R-soil and N-soil were T3 > CK > T2 > T1 and T3 > T2 > T1 = CK, respectively.

**Table 1:** Estimated OTU richness and diversity indices of the 16S rRNA gene libraries for clustering at 97% identity, as obtained from the pyrosequencing analysis.

Treatments	Total Tags	Taxon Tags	OTUs(97%)	Chao index	Shannon index
RCK	14347a	8616a	531a	550.47ab	7.32a
RT1	10277b	6027b	502a	486.65b	7.39a
RT2	13577a	8653a	516a	543.24ab	7.03b
RT3	15116a	9559a	534a	590.44a	7.21ab
NCK	11170b	6076b	500b	499.56b	7.55a
NT1	10863b	6552b	500b	490.78b	7.41a
NT2	19013a	11400a	549a	523.00ab	7.37a
NT3	9014b	5251b	489b	554.64a	7.43a

Total Tags: refers to the total number of collated sequences in the filter; Taxon Tags: refers to the number of Tags used to construct OTUs and obtain classification information; OTUs: refers to the number of OTUs finally obtained; RCK: R-soil in CK; RT1: R-soil in T1; RT2: R-soil in T2; RT3: R-soil in T3; NCK: N-soil in CK; NT1: N-soil in T1; NT2: N-soil in T2; NT3: N-soil in T3; Means followed by different letters are significantly different at p < 0.05; The same below.

Alpha diversity indices, including the Chao and Shannon indices, are widely used to analyse the richness and diversity of bacterial communities in a sample^23^. The Chao and Shannon indices of soil bacteria differed largely between different tetracycline addition treatments. The Chao index of soil bacterial species was decreased in the T1 treatment and increased in the T3 treatment when compared to the index in CK-treated R-soils. It was highest in the T3 treatment and lowest in T1 treatment with a significant difference between the two (p < 0.05). The changes in the Chao index between different treatments of N-soil had followed a trend similar to that of R-soil index changes. The Chao index in each treatment of R-soil was greater than that of N-soil.

The results of the Shannon index showed that the soil bacterial diversity was highest in CK treatment, followed by the T1 and T3 treatment. The lowest Shannon index was in the T2 treatment of R-soil or N-soil, which indicated that the effects of different amount of tetracycline addition on the bacterial diversity of R-soil were different, and the medium amount of tetracycline addition (50 mg/kg) had the greatest impact on soil bacterial diversity. The Shannon index in each treatment of N-soil was higher than that of R-soil, which might be related to the rhizosphere effect of flue-cured tobacco.

### Changes in the major groups and relative abundance of soil bacteria

**Figure 1:**
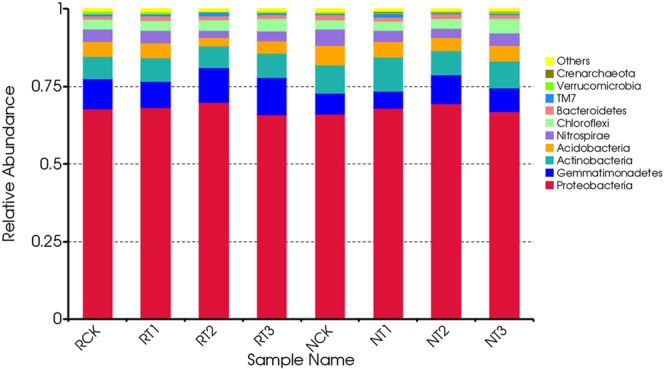
Changes in the abundance of soil bacteria at the phylum level in different treatments.

According to the results of OTU species annotation, ten dominant relative abundances of soil bacteria at the phylum level were shown in Fig. 1. Bacteria in the tetracycline-treated soil samples mainly included *Proteobacteria, Gemmatimonadetes, Actinobacteria, Acidobacteria, Nitrospirae, Chloroflexi, Bacteroidetes, TM7, Verrucomicrobia*, and *Crenarchaeota*. *Proteobacteria* had the highest relative abundance in soil samples, accounting for more than 60%. The relative abundances of *Bacteroidetes, Actinobacteria, Actinobacteria*, and *Nitrospirae* were also very high.

The relative abundance of *Proteobacteria* and *Gemmatimonadetes* were both highest in the T2 treatment of R-soil or N-soil, while the relative abundances of *Acidobacteria* and *Nitrospirae* were lowest. Similarly, the relative abundance of *Bacteroidetes* was slightly increased in R-soil samples but decreased in N-soil samples as the amount of tetracycline increased. Therefore, the relative abundance of bacteria at the phylum level might be related to the bactericidal action of tetracycline and its dose-effect relationship. Long-term exposure to high tetracycline stress could result in bacterial resistance and increase of antibiotic-resistant bacteria within the microflora.

**Figure 2:**
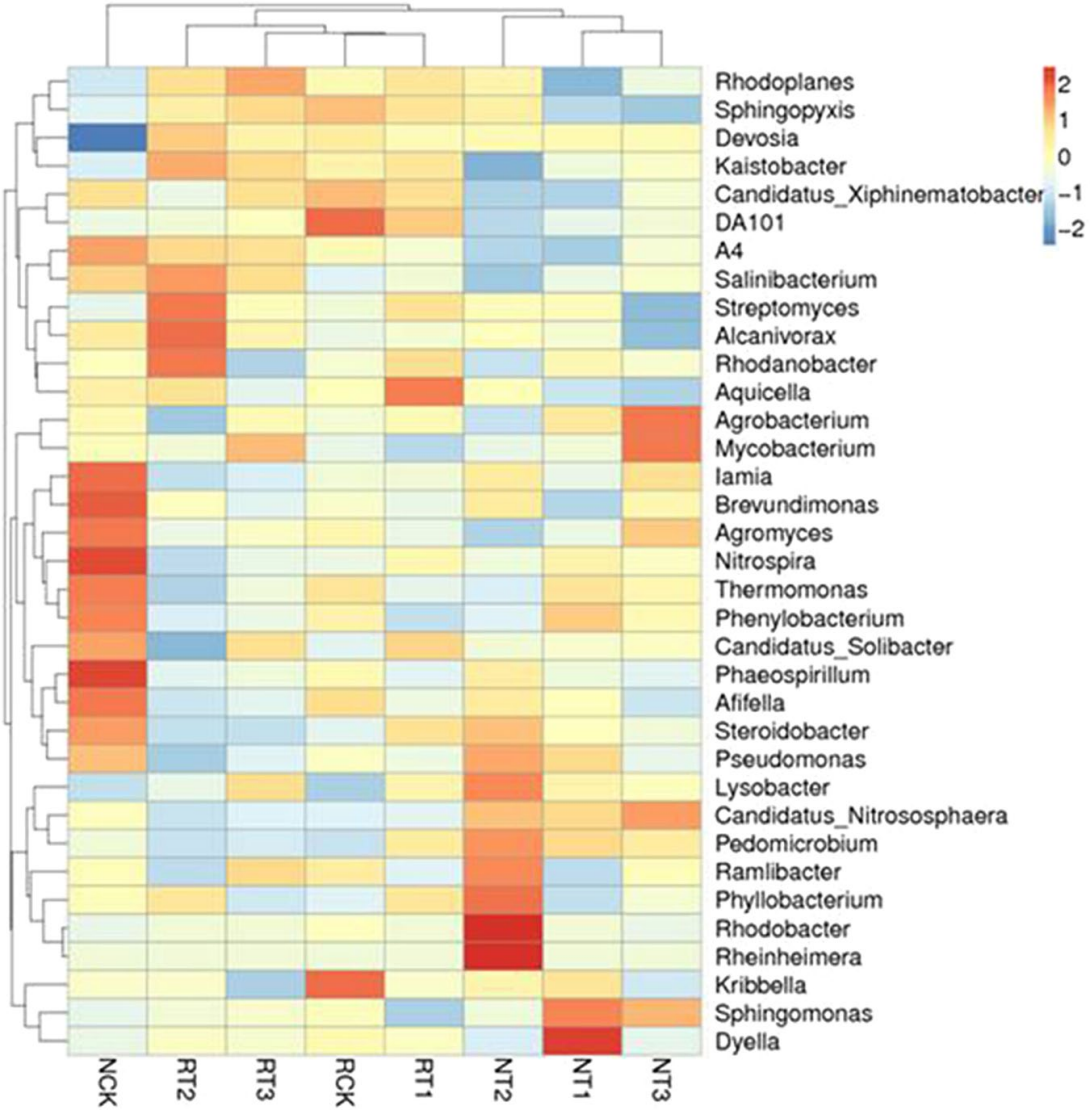
Species abundance clustering diagram of soil bacteria at the genera level in different treatments.

The heatmap of the domain top 35 soil bacterial genera in R-soils and N-soils was shown in Fig. 2. The genera of CK, T1, T2, and T3 treatments in R-soil were clustered together, which indicated that the soil bacterial community structure among R-soil treatments was similar. The genera of T1, T2, and T3 treatments of N-soil were clustered together, and the CK treatment of N-soil was far away from other treatments, indicating that tetracycline addition could significantly affect the structure of the soil bacterial community.

### Results of bacterial community clustering analysis of soil samples

**Figure 3:**
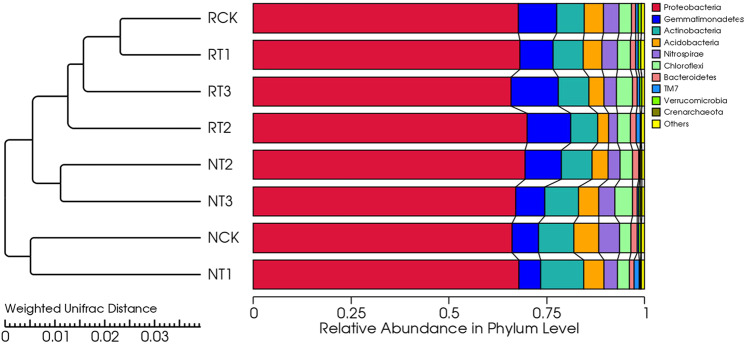
Clustering tree of soil bacteria at the phylum level based on Weighted Unifrac distance.

The soil samples were clustered and integrated with the relative abundance of soil bacteria species at the phylum level (Fig. 3). The cluster plot showed that all treatments were divided into two clusters. The CK and T1 treatments of N-soil were clustered together, indicating a similar community structure between them. All treatments of R-soil, as well as the T2 and T3 treatments of N-soil were clustered together, indicative of community structure similarities. The bacterial community pattern in R-soil was obviously different from that in N-soil, even though the affinity for certain bacterial communities showed some consistency within R-soil or N-soil. The different phylogenetic relationships of soil bacteria might be related to the compound effect of tetracycline addition and tobacco roots.

### Analysis of high-throughput sequencing results and alpha diversity of the fungal community

The preliminary statistical analyses were performed on total tags and taxon tags data of different samples during the OTU construction process (Table 2). The results revealed that an average of 51,210 total tags and 1192 OTUs per sample were obtained. The number of fungal sequences and OTUs in CK was significantly higher than those in other treatments of R-soil, while the lowest ones were found in the T3 treatment. Furthermore, the highest number of fungal sequences was found in T1, while the smallest was in T2 amongst all treatments of N-soil. However, the OTUs were different from the fungal tags in these treatments.

**Table 2:** Estimated OTU richness and diversity indices of the 18S rRNA gene libraries for clustering at 97% identity, as obtained from the pyrosequencing analysis.

Treatments	Total Tags	Taxon Tags	OTUs(97%)	Chao index	Shannon index
RCK	87107a	82515a	1684a	1599.57a	6.91a
RT1	46511b	44867b	1215b	1185.52b	5.41b
RT2	46918b	45256b	1222b	1168.45b	4.61b
RT3	37233b	35351b	1228b	1206.65b	5.12b
NCK	48781a	46633a	1278a	1227.67ab	4.97ab
NT1	54239a	53084a	942b	973.67bc	2.69c
NT2	37161b	34958b	1187ab	1632.37a	6.56a
NT3	51730a	50565a	778b	758.25c	3.67bc

Means followed by different letters are significantly different at p < 0.05.

The Chao and Shannon indices of soil fungal communities were shown in Table 2. The Chao and Shannon indices were both decreased and then increased with the amount of tetracycline addition in R-soil. Further, they were significantly lower in tetracycline addition treatments than in CK treatment. The Chao and Shannon indices in all treatments of N-soil were lower than those of R-soil except for T2. Therefore, the highest fungal community diversity might be maintained at intermediate levels of tetracycline disturbance in tobacco non-rhizosphere soil.

### Changes in the major groups and relative abundance of soil fungi

**Figure 4:**
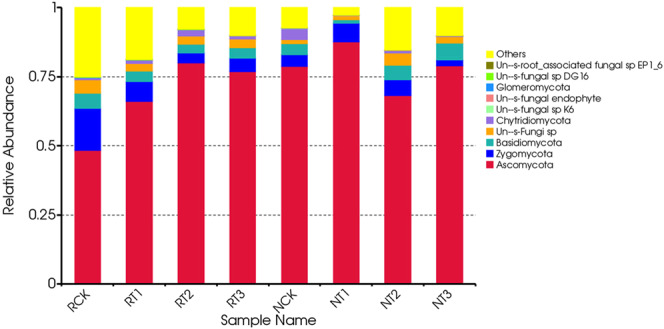
Changes in the abundance of soil fungi at the phylum level in different treatments.

The dominant relative abundances of ten phyla of soil fungi were selected to generate a column diagram of different treatments (Fig. 4). Fungi in the tetracycline treated soil samples included *Ascomycota, Zygomycota, Basidiomycota, Un-s-Fungi sp* (unidentified), *Chytridiomycota, Un-s-fungal sp K6, Un-s-fungal endophyte, Glomeromycota, Un-s-fungal sp DG 16*, and *Un-s-root-associated fungal sp EP 1-6*. The relative abundance of *Ascomycota* was more than 60% in all treatments except for CK treatment of R-soil.

In the R-soil samples, the relative abundance of major soil fungi changed with the amount of tetracycline addition at the phylum level. The relative abundance of *Ascomycota* decreased in the order of RT2 > RT3 > RT1 > RCK, but the *Zygomycota* and *Basidiomycota* had an opposite trend, and the *Un-s-fungi sp* gradually decreased in the order of RCK > RT3 > RT1 > RT2. In N-soil samples, the relative abundances of *Ascomycota*, *Zygomycota*, *Basidiomycota*, and *Un-s-fungi sp*. in different treatments varied with the amount of tetracycline addition. The relative abundance of other fungi in all treatments in R-soils was greater than that in the N soils, except for T2, which showed opposite results.

**Figure 5:**
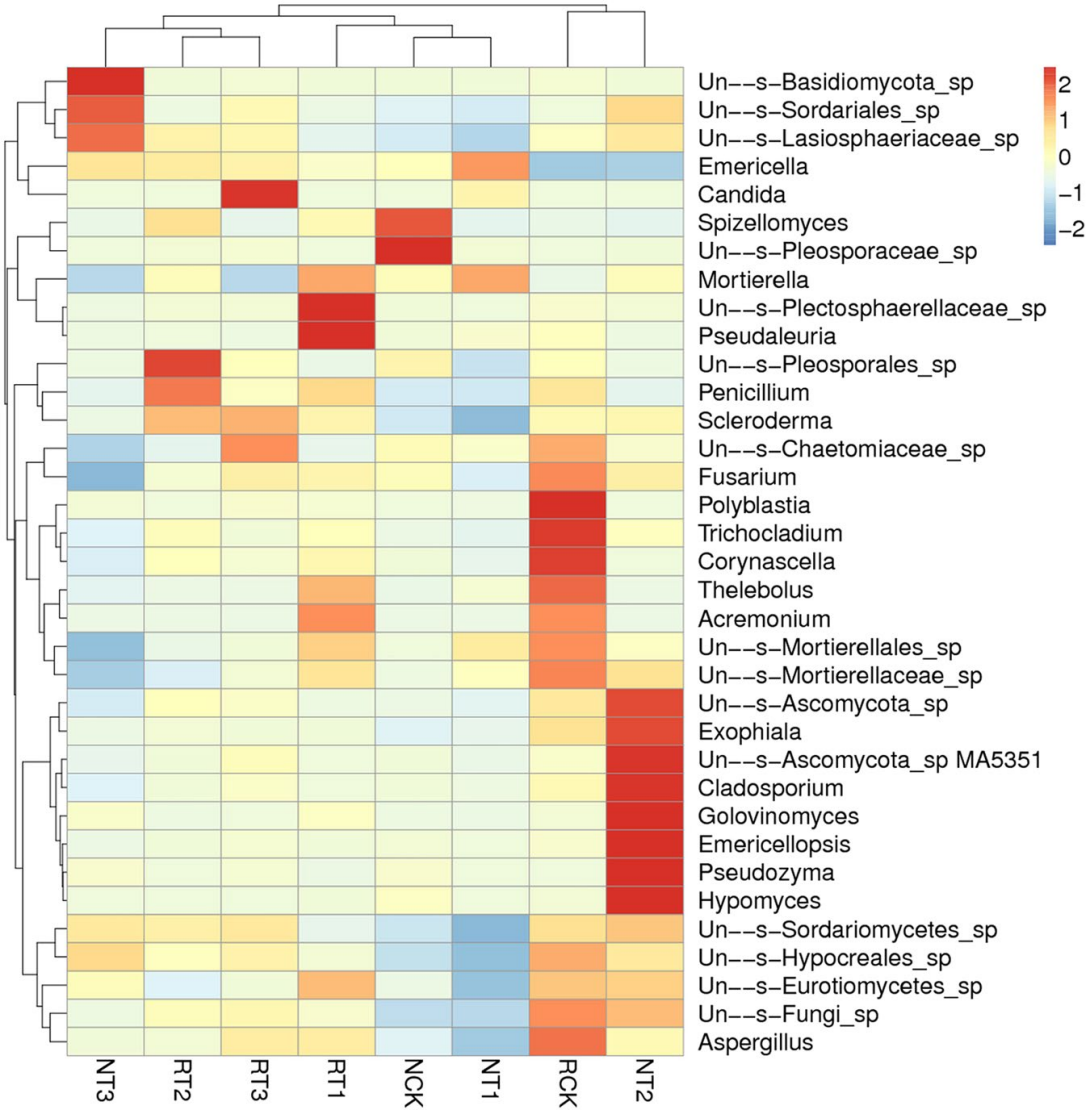
Species abundance clustering diagram of fungi at the genera level in different treatments.

As shown in Fig. 5, the heat map of the top 35 soil fungal genera in each soil sample was constructed in order to identify the similarities and differences of fungal community structures. All treatments were divided into three clusters. The genera in T2 and T3 treatments of R-soil and T3 treatment of N-soil were clustered together. CK and T1 treatment of N-soil and T1 treatment of R-soil were also clustered together. CK treatment of R-soil and T2 treatment of N-soil were clustered close to one another, which also implied similarities between fungal communities.

### Results of soil fungal community clustering analysis

**Figure 6:**
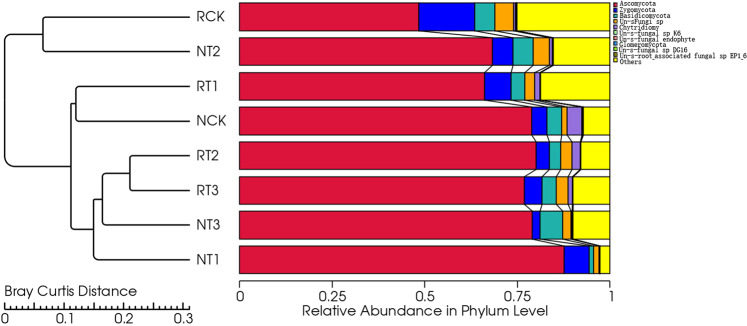
Clustering tree of soil fungi at the phylum level based on Bray-Curtis distance.

The clustering tree of soil fungi at the phylum level with different treatments based on Bray-Curtis distance was plotted in Fig. 6. All treatments were divided into two clusters. The CK treatment of R-soil and T2 treatment of N-soil were clustered together, indicating a similar community structure between them. The T2, T3 treatments of R-soil, and T1, T3 of N-soil, were also clustered together, indicative of similar fungal communities. Additionally, the two clusters were well separated from each other, which implied clear distinctions of fungal community structure between them.

## Discussion

In the current study, high-throughput sequencing technology was used to analyse the effects of tetracycline residues on microbial community structure in tobacco soil. The results showed that soil bacterial community richness and diversity as well as the relative abundance of main bacterial populations all changed with tetracycline addition. The bacterial richness of R-soil and N-soil showed a consistent trend, which decreased and then increased with the amount of tetracycline addition. High concentrations of tetracycline could continuously act on soil bacteria, thereby inducing the emergence of antibiotic-resistant bacteria^24,25^. Tetracycline has a broad spectrum of bactericidal properties that can inhibit or kill certain types of soil bacteria, resulting in reduced bacterial diversity^26,27^. However, higher tetracycline concentrations could provide C sources for the surviving bacteria, induce the generation of drug-resistant bacteria, and thus increase soil bacterial diversity^7,28^. The bacterial Chao indices for all treatments of R-soil were higher than those in N-soil, and the Shannon indices had the opposite trend, which might be related to the continuous changes in the local environment of root exudates^29,30^ and surrounding soil^31^. Li *et al*. (2012) also found that the Shannon index of non-rhizosphere soil was higher than that of rhizosphere soil at the mature tobacco stage at three sampling sites^32^. Rhizosphere is an important site of substance and energy exchange between plants and soil microbes. The soil microbial diversity in rhizospheres has consistently been described as higher than that in non-rhizosphere of tobacoo^33^, which is similar to other crops. The soil bacterial diversity in the rhizosphere of medicinal herbs was found to be higher than in bulk soil, which was not shown to be significant according to the Chao index and Shannon index^34^. Rice rhizosphere soils displayed higher bacterial diversity indices than bulk soils contaminated with mixed heavy metals^35^. Therefore, the tobacco root exudates would probably stimulate soil microbes to proliferate and metabolise, resulting in higher OTUs and diversity of the soil microbial community in tobacco rhizosphere soil (Table 1 and Table 2). Soil microbial communities (bacteria and fungi) were strongly determined by the biogenic resource parameter^36^.

In the current study, *Proteobacteria, Bacteroidetes, Actinobacteria, Acidobacteria, Nitrospirae, Chloroflexi, Bacteroidetes, TM7*, and *Verrucomicrobia* were the main types of bacteria in the soil (Fig. 3). Tetracycline addition could change the microbial community composition by altering the relative abundances of dominant bacteria. Treatment-induced differences in the relative abundance of bacteria were mainly due to two reasons; (i) the inhibition and bactericidal effect of tetracycline, (ii) the induction of bacterial antibiotic resistance. After tetracycline is applied to the soil, a series of degradation reactions occur, including biodegradable and non-biodegradable reactions^37^. The biodegradation processes include microbial degradation and plant degradation^26^. The non-biodegradation processes include photo-degradation, oxidative degradation, and hydrolysis. Environmental conditions also influence the tetracycline degradation in soils. Tetracycline has a half-life of 14.1–69.3 days^38^, but the half-lives of its degradation products could be as long as 400 days in soil interstitial water^39^. Some degradation products are as potent as tetracycline^40^. These recalcitrant metabolites remain bioactive and continually exert selective pressure on soil microbes, and thus might be responsible for the persistence of antibiotic resistance genes (ARGs) in soils, even though the parent compounds have been depleted. High concentrations of tetracycline would continue to act, resulting in differences of bacterial community structure in the soil. Li *et al*. (2019) found that the abundance of ARGs was increased with the concentrations of tetracycline in soils^41^, which might have resulted from the induction of high selective pressure by antibiotics.

Apart from soil bacterial diversity, tetracycline addition had effects on the fungal community structure of tobacco soil. The soil fungal community structure in tetracycline-treated soil was different from that of CK treatment. Chao and Shannon indices in all tetracycline-treated soils were much lower than those in their CK-treated counterparts except for T2 in N-soil. The negative impact on soil fungal community was presumably associated with the suppression of sensitive species by tetracycline addition. Several studies found that different biocides have different impacts on off-target microbial communities, depending on soil physicochemical properties, the amount applied, and the biocide type^42–44^. Tetracycline addition had a negative effect on off-target fungi communities in tobacco soil in this study (Table 2). Similarly, soil treatment with oxytetracycline dosages could also negatively affect the growth of off-target soil fungi^45^ and the Shannon diversity index of soil microbial community^46^. Changes in the structure of fungal communities might be the result of interactions between fungi and bacteria in the soil under tetracycline addition. Studies have shown that some fungi in the rhizosphere are affected by surrounding bacteria during plant growth^47,48^. The impact would be positive, neutral, or negative. The fungal-bacterial associations could affect the composition of the surrounding matrix in the soil through physical, biological, and biochemical processes. Many bacteria and fungi usually occupy a common micro-habitat, thus comprising bacterial-fungal interface^49^. In the interface, organisms are ecologically neutral (inactive), they compete with and antagonise or, alternatively, cooperate with each other. Thus, the interaction between these two partners in the interface might vary depending on their ecological physiology and local soil conditions^50,51^. As reported, tetracycline did not influence the fungal population but clearly stimulated the actinomycete bacteria, antibiotic-producers, other soil microflora, and thus influenced the composition of the soil microbial community^41,52^.

In the current study, T2-treated soil had the highest fungal community diversity (Chao and Shannon indices) among all tetracycline treatments of N-soil, which might be associated with the growth of bactericide-tolerant fungi or decreased competition of bacteria inhibited by tetracycline stress at medium concentrations^53^, a response that was consistent with the intermediate disturbance hypothesis^54^. Most fungi appeared to be more resistant to environmental stress than bacteria in bulk soil. Because fungi have more sophisticated mechanisms of dispersal that do not exist in bacteria^55^, this could have helped them to fill the vacant soil niches opened from the intermediate disturbance. Moreover, considering the higher complexity of fungal genomes, it is possible that many possess a niche breadth larger than those allowed by bacterial genomes, and thus, their community diversity might be greater under intermediate disturbance^56^. Therefore, the response of soil fungal and bacterial community diversity to different concentrations of tetracycline is a complex process, the inner mechanisms and driving factors of which, still need to be further explored.

## Conclusions

High-throughput sequencing technology was used to study the effects of different concentrations of tetracycline on the microbial community structure of tobacco soil in pot experiments. The results showed that tetracycline addition had an important effect on the richness, diversity, and structure of soil bacterial and fungal communities. High tetracycline addition continuously acted on soil bacteria and induced evident concentration-dependent effects. There was a similar tendency in the effects of tetracycline on soil bacterial community diversity in the rhizosphere and non-rhizosphere soil of tobacco. The bacterial colonies in the rhizosphere with higher diversity were closely clustered into one group, which might be the result of tobacco root secretions and rhizodeposition. The effects of tetracycline on soil fungal community structure were influenced by bacterial–fungal interactions, and the intermediate disturbance (50 mgTc kg^−1^) significantly increased the soil fungal diversity in the non-rhizosphere of tobacco, indicating that the specific effect of tetracycline on soil fungal community needs to be further studied in greater detail. In addition, the results of this study were based on a single sampling, which could characterise the changes only in a time slice. In the future, dynamic monitoring of the tobacco soil micro-ecological environment, treated with different concentrations of tetracycline, should be carried out. This would be of great importance to further our understanding of the effects of tetracycline on soil microbial dynamics.
